# Evaluation of Functional Capacity and Satisfaction of Patients With Lumbosacral Fusion

**DOI:** 10.7759/cureus.34284

**Published:** 2023-01-27

**Authors:** Ömer Bozduman, Ömer Cahit Çıtır, Enes Gürün

**Affiliations:** 1 Orthopaedic Surgery, Samsun University Faculty of Medicine, Samsun, TUR; 2 Orthopaedic Surgery, Şırnak State Hospital, Şırnak, TUR; 3 Radiology, Samsun University Faculty of Medicine, Samsun, TUR

**Keywords:** visual analog scale (vas), low back pain, pittsburgh sleep quality ındex (psqı), oswestry scale, visual analogue scale, lumbosacral fusıon

## Abstract

Introduction: Spinal fusion is a surgical procedure that has been successfully conducted for many years. It is applied for various indications, such as degeneration, deformity, instability, spinal stenosis, trauma, tumor, and infection. This study aims to determine the effects of this procedure on daily life and sleep quality by examining postoperative symptoms and refractory complaints of patients who underwent lumbosacral fusion for various indications.

Methods: The files of the patients who underwent only posterolateral lumbosacral fusion for various indications between June 2021 and July 2022 were reviewed retrospectively. Patients who had had regular clinical follow-ups for at least six months postoperatively were included in the study. Preoperative and postoperative Visual Analog Scale (VAS), Oswestry Disability Index (ODI), and Pittsburgh Sleep Quality Index (PSQI) scores were compared using the Wilcoxon Ordinal Signs test. A p-value of <0.05 was considered statistically significant.

Results: Twenty patients were included in the study. The mean age of the patients was 68.2 ± 7.5 (54-79). Three (15%) of the patients were males, and seventeen (85%) were females. Improvement was observed in all three scores, i.e., VAS, ODI, and PSQI assessments. No correlation was found between the number of segments undergoing fusion, body mass index (BMI), and clinical outcomes.

Conclusion: Spinal fusion surgery is still viewed as the gold standard treatment method for many indications. Posterolateral fusion provides adequate stabilization in many cases when applied correctly. However, the possibility of persistent or newly developing low back pain in the postoperative period as a result of mechanical reasons should not be forgotten, and patients should be informed about the same. Postoperative expectations should, thus, be shaped accordingly.

## Introduction

Spinal fusion is a surgical procedure that has been successfully conducted for many years [1]. It is applied for various indications, such as degeneration, deformity, instability, spinal stenosis, trauma, tumor, and infection [2-4]. The purpose of fusion surgery is to eliminate pain caused by a dysfunctional intervertebral disc structure. In this procedure, the vertebral segments that cause pain when they move are fixed, a reduction of movement is achieved, and the load distribution is balanced. This surgery aims to provide a stable reduction movement, neurological decompression and fusion, and ultimately, pain treatment [1].

Although the indication and treatment protocols for the lumbosacral region are similar to those for the lumbosacral junction, the latter has some unique features [5]. The sacrum is almost immobile as a result of the rigid connections between the sacrum and the iliac wings. In contrast, the lumbar vertebral column is mobile [5]. The results of fusion surgeries to be performed at this level, which is a transition zone in terms of anatomical and mechanical properties, are likely to be different from other levels.

The literature has shown that sacroiliac syndrome may develop due to increased tension in the sacroiliac joint, which develops as a result of the changed mechanical structure. As a result of lumbosacral fusion, gluteal tendinitis may develop due to changing loads, and sacral stress fractures may occur as a result of the distribution of these non-physiological forces [6-8]. This study aims to determine the effects of this procedure on daily life and sleep quality by examining postoperative symptoms and refractory complaints of patients who underwent lumbosacral fusion for various indications.

## Materials and methods

The data of patients who underwent lumbosacral fusion for various indications in Samsun Education and Research Hospital, Samsun, Turkey, between June 2021 and July 2022 were reviewed retrospectively. The study was approved by the Samsun University Clinical Research Ethics Committee (Approval number: 2023/1/6). Patients who had at least six postoperative months and regular clinical follow-ups were included in the study. Patients with symptoms related to known lumbar pathology, those with late surgical complications (e.g., infection or implant failure), and those with symptomatic inflammatory, tumoral, or traumatic issues were excluded from the study. In addition, patients who had hip arthroplasty before or after lumbosacral fusion or coxarthrosis were not included in the study. Twenty patients who met these criteria were selected and included in the study.

**Figure 1:**
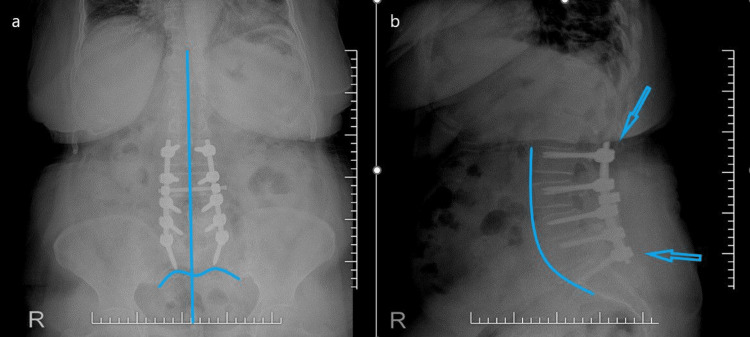
Posterior lumbosacral instrumentation (a) anteroposterior and (b) lateral digital radiographs. The blue vertical line shows the central sacral vertical line, the blue curved line shows the lumbar lordosis, and the arrows show the pedicle screws and rods.

All of the patients included in the study underwent decompression and posterior instrumentation. Demographic values such as age, height, weight, and body mass index (BMI) were documented. The number of segments included in the fusion was recorded. Routine anteroposterior and lateral radiographs of the patients were evaluated and show the lumbar lordosis for sagittal balance and central sacral vertical line for coronal balance (Figure 1).

In addition, the Visual Analogue Scale (VAS), Oswestry Disability Index (ODI), and Pittsburgh Sleep Quality Index (PSQI) evaluations were used to evaluate the clinical results and sleep quality of all patients included in the study.

The IBM SPSS Statistics for Windows, Version 21.0 (Released 2012; IBM Corp., Armonk, New York, United States) was used for statistical analysis. The mean, standard deviation, minimum, and maximum values were used to present the descriptive data. The distribution of variables was measured with the Kolmogorov-Smirnov test. The pre- and postoperative VAS, ODI, and PSQI scores were compared with the Wilcoxon Ordinal Signs test. The relationship between the pre- and postoperative changes in VAS, ODI, and PSQI scores, along with BMI and the number of segments that underwent fusion, was investigated using the Spearman correlation test. A p-value of <0.05 was considered statistically significant.

## Results

Twenty patients were included in the study. The mean age of the patients was 68.2 ± 7.5 (54-79) years. Three patients (15%) were male, and 17 (85%) were female. Three-level fusion was applied to six patients (30%), four-level to five (25%), five-level to eight (40%), and one patient had six or more levels of fusion. In terms of BMI, six patients (30%) were of normal weight (BMI 18.5-24.9), 10 patients (50%) were overweight (BMI 25-29.9), and four (20%) were obese (BMI ≥ 30).

In the clinical control interview, when asked to compare their preoperative condition to their current condition, the average of the VAS scores determined in the preoperative period was 8.1 ± 0.8 (7-9), and the mean VAS score in the postoperative period was 3.6 ± 1.1 (2-6). The positive change in the VAS scores was statistically significant (p < 0.001).

The ODI was used to determine the level of effect of clinical symptoms on daily quality of life and functions. Patients were asked to evaluate the preoperative period and their current state. Preoperatively, two patients (10%) were completely limited in their daily life, 13 patients (65%) were severely limited, and five patients (25%) were mildly limited. In the postoperative period, no patient reported complete limitation; four patients (20%) had severe limitation, seven patients (35%) reported mild limitation, and nine patients (45%) did not have significant limitations. It was determined that the limitation experienced by the patients in daily life due to low back pain had decreased substantially; the positive changes in ODI scores were statistically significant (p < 0.001).

Similarly, the results of the PSQI, which was applied to determine the effect of clinical symptoms on sleep quality, were 5.95 ± 2.70 in the preoperative period and 3.60 ± 1 in the postoperative period. This improvement in PSQI scores following surgery was statistically significant (p < 0.001), and the number of patients reporting good sleep quality also increased significantly.

The correlation of the number of fused segments with changes in VAS score (p = 0.661, rho = -0.105), ODI score (p = 0.562, rho = 0.562), and PSQI score (p = 0.211, rho = 0.292) was not statistically significant. Similarly, the correlation of body mass index with VAS score (p = 0.707, rho = -0.090), ODI score (p = 0.277, rho = -0.255), and PSQI score (p = 0.656, rho = 0.106) was not statistically significant.

## Discussion

The aim of this study was to evaluate the clinical satisfaction and functional abilities of patients who underwent lumbosacral fusion. The results showed that the VAS score decreased, functional ability increased according to the ODI score, and sleep quality increased according to the PSQI score. However, using the VAS and ODI scales, it was found that there were patients with persistent pain and functional limitations. From this point of view, it was considered that the expectations of patients who underwent lumbosacral fusion should be properly understood in terms of the postoperative period, and detailed information about the possible clinical outcomes should be provided. It was considered that it is important for the patient to be aware of the possibility of refractory retention or the development of new symptoms in order to make a surgical decision with more realistic expectations.

Fusion surgery, which is applied in cases of various indications, such as degeneration, deformity, instability, spinal stenosis, trauma, tumor, and infection, is still seen as the gold standard treatment method [1,2]. Nevertheless, complaints of pain often persist despite surgery or recur after surgery. In current literature, the source of this pain is reported to be the sacroiliac joint [9-11]. Theories regarding the mechanism of pain suggest ligament or capsule stretching, imbalance of loading or shear forces, hypomobility or hypermobility, abnormal changes in joint mechanics, and inflammation [12].

In line with the literature, we observed that 20 patients examined in our study had persistent low back pain during their clinical follow-up. Statistically significant improvement was observed according to the VAS and ODI scores. Many studies in the literature have shown that lumbosacral fusion surgery has a positive effect on clinical scores [13-15]. The findings from this study support the literature. However, it was considered noteworthy that the mean VAS value of the patients could regress to a level close to moderate pain (3.6), and that 20% similarly had severe limitations in daily life, according to the ODI score, in the postoperative process.

Considering that all the factors thought to cause pain after lumbosacral fusion are mechanically based, it is possible to have a positive improvement in the PSQI score in the postoperative period. While the mean PSQI value was determined to be 5.95 ± 2.70 in the preoperative period, the value was 3.60 ± 1.6 in the postoperative period. This improvement was statistically significant (p < 0.001). In this patient group, it was thought that the study could be instructive since no other study in the literature had evaluated PSQI.

When the effect of the number of fusion segments was examined, no statistically significant difference was observed. There are publications in the existing literature that report different results on this subject. Ivanov et al. and Unoki et al. reported that the number of segments undergoing fusion adversely affects clinical results and causes sacroiliac syndrome [16,17]. In contrast, this correlation could not be demonstrated in a study published by Ha et al. [18].

It should be noted that the results obtained may be affected by parameters such as age, physical activity expectation, and osteoporosis, taking into account that the lumbosacral region is under significant mobility and torsional forces caused by the weight of the trunk. As a result of the loss of mobility following lumbosacral fusion, an increase in the lordosis of the more proximal vertebrae develops, aimed at preserving normal lordosis. As a result of this compensatory movement, increased stress on the facet joints may cause pain [19]. In distal fusion, pelvic tilt is increased in order to prevent pelvic retroversion, and pain may develop in the lower extremities as a result of the deterioration in the balance of the sacral slope and pelvic index [19].

Our main limitations were the retrospective nature of the study and the absence of a control group in which the sacrum was not included in the fusion. Other limitations are the small number of patients, the short follow-up period, and the fact that the patients who underwent fusion comprised posterolateral fusion patients who underwent only posterior instrumentation.

## Conclusions

In this study, it was found that in patients who underwent lumbosacral fusion, the VAS score decreased, functional capacity increased according to the ODI score, and sleep quality increased according to the PSQI score. However, using the VAS and the ODI scales, it was found that there were patients whose pain and functional limitations persisted. At the same time, it was found that the number of fused segments and the BMI value of the patients had no statistically significant effect on clinical outcomes.
